# The Developmental Toxicity of Complex Silica-Embedded Nickel Nanoparticles Is Determined by Their Physicochemical Properties

**DOI:** 10.1371/journal.pone.0152010

**Published:** 2016-03-31

**Authors:** Sharlee Mahoney, Michelle Najera, Qing Bai, Edward A. Burton, Götz Veser

**Affiliations:** 1 Department of Chemical Engineering, University of Pittsburgh, Pittsburgh, Pennsylvania, United States of America; 2 Mascaro Center for Sustainable Innovation, University of Pittsburgh, Pittsburgh, Pennsylvania, United States of America; 3 Department of Neurology, University of Pittsburgh, Pittsburgh, Pennsylvania, United States of America; 4 Pittsburgh Institute for Neurodegenerative Diseases, University of Pittsburgh School of Medicine, Pittsburgh, Pennsylvania, United States of America; Brandeis University, UNITED STATES

## Abstract

Complex engineered nanomaterials (CENs) are a rapidly developing class of structurally and compositionally complex materials that are expected to dominate the next generation of functional nanomaterials. The development of methods enabling rapid assessment of the toxicity risk associated with this type of nanomaterial is therefore critically important. We evaluated the toxicity of three differently structured nickel-silica nanomaterials as prototypical CENs: simple, surface-deposited Ni-SiO_2_ and hollow and non-hollow core-shell Ni@SiO_2_ materials (i.e., ~1–2 nm Ni nanoparticles embedded into porous silica shells with and without a central cavity, respectively). Zebrafish embryos were exposed to these CENs, and morphological (survival and malformations) and physiological (larval motility) endpoints were coupled with thorough characterization of physiochemical characteristics (including agglomeration, settling and nickel ion dissolution) to determine how toxicity differed between these CENs and equivalent quantities of Ni^2+^ salt (based on total Ni). Exposure to Ni^2+^ ions strongly compromised zebrafish larva viability, and surviving larvae showed severe malformations. In contrast, exposure to the equivalent amount of Ni CEN did not result in these abnormalities. Interestingly, exposure to Ni-SiO_2_ and hollow Ni@SiO_2_ provoked abnormalities of zebrafish larval motor function, indicating developmental toxicity, while non-hollow Ni@SiO_2_ showed no toxicity. Correlating these observations with physicochemical characterization of the CENs suggests that the toxicity of the Ni-SiO_2_ and hollow Ni@SiO_2_ material may result partly from an increased effective exposure at the bottom of the well due to rapid settling. Overall, our data suggest that embedding nickel NPs in a porous silica matrix may be a straightforward way to mitigate their toxicity without compromising their functional properties. At the same time, our results also indicate that it is critical to consider modification of the effective exposure when comparing different nanomaterial configurations, because effective exposure might influence NP toxicity more than specific “nano-chemistry” effects.

## Introduction

Nanomaterials are about to fundamentally alter how we exploit the chemical and physical properties of materials. This raises the possibility that unexpected nano-specific toxicity will occur through mechanisms that cannot be extrapolated from the analogous bulk material properties [1–4]. The size range over which reported nanotoxicity is greatest (d < 20 nm) [5] correlates with the range in which most of the unique and desirable properties of nanomaterials appear [6], and development and production of materials in this size range is rapidly accelerating [7]. Consequently, there is an urgent need to develop methods that allow for sensitive and high-throughput evaluation of nanomaterial toxicity.

In practice, nanoparticles (NPs) are rarely used as independent structures because they tend to aggregate and/or sinter, resulting in deactivation and loss of their desired nano-specific properties. To overcome deactivation, nano-enabled materials are often designed as multi-component materials which embed active NPs within a protective matrix [8, 9]. When these multicomponent nanomaterials are rationally designed in hierarchical nanostructures, they are often referred to as complex engineered nanomaterials (CENs). CENs are expected to dominate future nanomaterials. However, to date, few studies have been conducted on CENs, despite the observations that, compared to single component nanostructures, the toxicity of CENs can be enhanced [10, 11] or attenuated [12].

In order to guide the design of safer nanomaterials, a thorough characterization of the materials’ physicochemical properties is critical in order to identify robust structure-toxicity correlations. Size, shape, surface area, surface chemistry, aggregation, agglomeration, settling, and dissolution have all been identified as key material properties that can be indicators for nanotoxicity [13]. These material attributes are often difficult to characterize for CENs due to their increased structural and compositional complexity compared to single-component materials. A systematic variation of the nanoconfiguration of a CEN with defined composition, combined with a thorough physicochemical characterization, allows to break down this complexity and can hence offer a novel approach towards studying how structural properties affect toxicity. This can offer insights into the design of safer nanomaterials through specific nano-configurations that minimize toxic properties, and allows prioritizing high risk materials for further evaluation [5, 14].

Zebrafish (*Danio rerio*) provide a rapid, inexpensive, and well-characterized model to evaluate the toxicity of chemical structures in a vertebrate organism *in vivo* [12, 15, 16]. They are prolific breeders, producing between 50–100 offspring with each mating. Zebrafish embryos and larvae are small and can be housed in 96-well plates [17]. This provides a convenient and powerful format for high-throughput assays to evaluate the toxicity of different compounds rapidly and over a range of concentrations [18]. As a vertebrate, the zebrafish shares a common basic body plan with other vertebrates, including mammals, and many molecular mechanisms governing early embryogenesis are also shared [19–21]. Consequently, zebrafish assays may provide insights into the teratogenic potential of test compounds in humans [22, 23]. Furthermore, zebrafish develop rapidly and externally, allowing direct observation of survival and morphology, which can be employed as simple assay end-points in parallel with sensitive physiological measurements such as assays of neurological function [17, 22]. Zebrafish are also uniquely placed as a model to provide insights into the harmful effects of industrial discharges on aquatic life. In view of these advantages, zebrafish are already used to test the toxicity of chemicals and drugs [17, 24–26], including early life stage toxicity tests of ToxCast Phase I chemicals using high throughput assays such as survival and malformations [27]. More recently, zebrafish embryos and larvae have been used for nanotoxicological research, using simple and robust end points, such as mortality [28–32], hatching [15, 16, 33–39], malformation [40, 41]. Significantly, zebrafish larval motility has now been employed as an endpoint in several developmental nanotoxicity studies, and validated as a separate index of neurodevelopmental outcome [42–45]. In comparison with other vertebrate models, zebrafish studies are characterized by low cost [46], and high throughput, due to the prolific breeding potential of adult zebrafish and the ability to study a large number of animals in parallel with limited space requirements. Additionally, automated assays measuring neurobehavioral parameters can be carried out in samples of 96 animals simultaneously, substantially increasing the throughput in comparison with mammalian models. Zebrafish studies typically focus on the first five days post fertilization, during which there is rapid development from the single-cell stage to a free-swimming vertebrate with all major organogenesis complete and a repertoire of simple and complex behaviors. Together, these properties of the zebrafish model allow multiple concentrations of different toxicants to be evaluated quickly and in parallel, with functional and morphological end points relevant to vertebrate embryogenesis and physiology.

In the present study, we investigated the toxicity of well-defined nanostructured nickel-silica CENs. Nickel NPs are finding widespread applications in industrial catalysis [47–50] and are often supported on silica [51–53] to enhance reactivity and stability. However, nickel NPs have been shown to be toxic [31, 54] in both *in vivo* and *in vitro* models, while amorphous silica is generally reported to be non-toxic [55, 56]. Workers in chemical plants and the fuel processing industry are at risk for occupational exposure to nickel and silica CENs. It is therefore important to fully understand the toxicity associated with these materials [57, 58].

We selected three morphologies representing prototypical complex engineered nanomaterials: (i) materials in which nickel NPs are deposited on the external surface of a silica particle (Ni-SiO_2_), and (ii) hollow and (iii) non-hollow core-shell materials (hNi@SiO_2_ and nhNi@SiO_2_, respectively), which consist of nickel NPs embedded into (porous) silica shells. These materials are thus compositionally complex and rationally designed to have a well-controlled hierarchical structure and hence constitute prototypical CENs. We hypothesize that the structure and embedding of the nickel NPs in the amorphous silica will alter the CENs physicochemical properties and thus affect their toxicity. In order to test this hypothesis, these CENs were subjected to thorough physicochemical characterization, including the determination of dissolution, settling and aggregation properties. The toxicity of these materials was then evaluated in zebrafish larvae, using three complementary endpoints: survival, developmental morphology and motor function. The first two are considered robust, established assays in nanotoxicology, whereas motor function is a novel and recently emerging assay for evaluating nanotoxicity.

## Results

### Characterization of Ni/SiO_2_ CENs

#### CEN size and morphology

**Fig 1:**
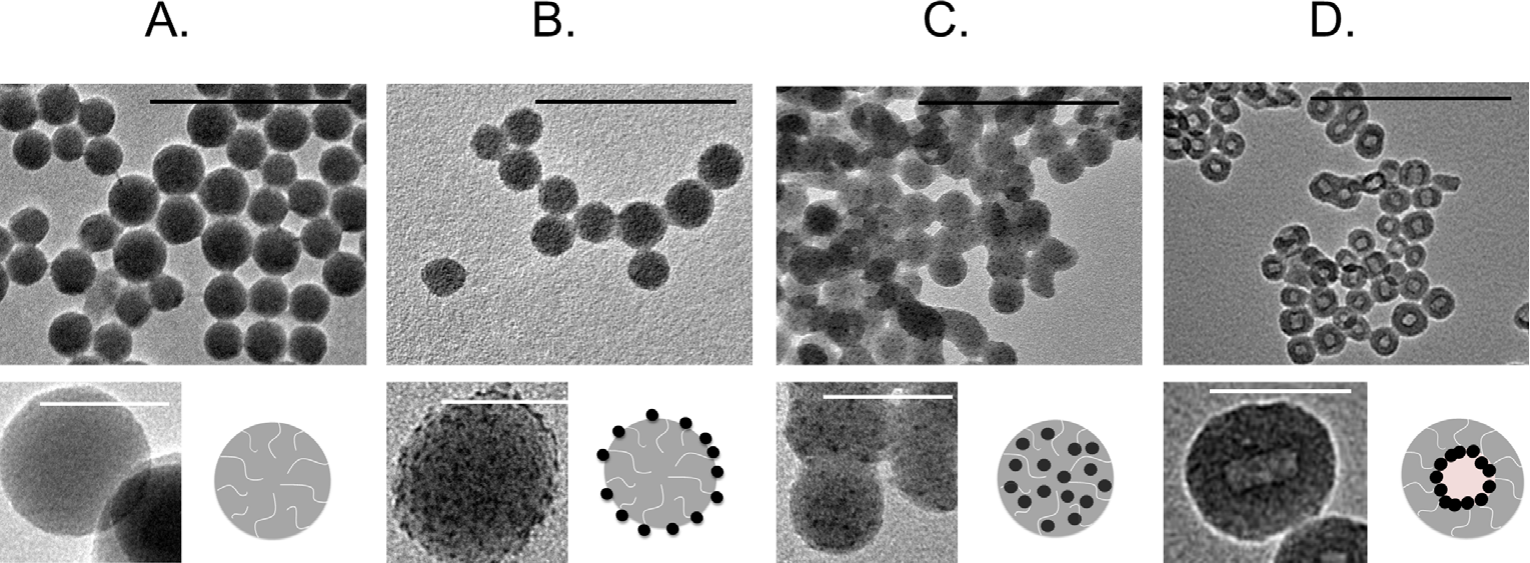
TEM images of typical (a.) metal-free SiO_2_, (b.) Ni-SiO_2_, (c.) nhNi@SiO_2_ and (d.) hNi@SiO_2_ samples. Scale bars are 200 nm for larger images (black) and 50 nm for smaller pictures (white).

Transition electron microscopy (TEM) images of the Ni-containing CENs are shown in Fig 1, and material characteristics are summarized in Table 1. For all three CENs, the nickel NPs are similar in size (~1–2 nm) and nickel content is ~9–12 wt%. The composite NPs are spherical and ~40–55 nm. The silica porosity and surface area are also similar, with surface areas of ~200–300 m^2^/g and average silica pore diameters of ~0.7 nm (S1 Fig). The silica microstructure is amorphous and the embedded nickel particles are crystalline (S2 Fig). All three materials share common chemical compositions and have similar dimensions, and differ in nanostructure alone. Occasional necking between adjacent particles in TEM indicates the presence of some aggregation in the synthesized material (likely due to formation of oxygen bridges during thermal treatment). To assess agglomeration in the zebrafish E3 medium (49 mM NaCl, 1.6 mM KCl, 3.3 mM CaCl_2_^.^, 3.3 mM MgSO_4_^.^, pH 7.4), CENs were dispersed in E3 medium solution by sonication, and agglomerate sizes were measured using dynamic light scattering (DLS, Table 1). After dispersion in E3 media, all three CENs agglomerate to similar sizes.

**Table 1:** Ni loading (from EDX), Ni particle size (from TEM)(dry) CEN particle size (from TEM), and CEN agglomerate size suspended in E3 medium (from DLS) for hNi@SiO_2_, nhNi@SiO_2_, and Ni-SiO_2_ CENs shown in Fig 1.

Nanoparticle	Ni loading (wt% Ni)	D_p_, Ni particle (nm), n = # particles counted	D_p_, primary particle (nm), n = # particles counted	Mean size in E3 medium suspension, (nm)
hNi@SiO_2_	9.0 ± 0.9	< 2	43.26 ± 7.2, n = 105	301 ± 22
nhNi@SiO_2_	9.6 ± 0.4	2.5 ± 0.4, n = 55	41.2 ± 8.5, n = 102	292 ± 27
Ni-SiO_2_	11.9 ± 3.2	2.1 ± 0.4, n = 73	54.5 ± 8.2, n = 112	322 ± 77

#### Ni ion dissolution

**Fig 2:**
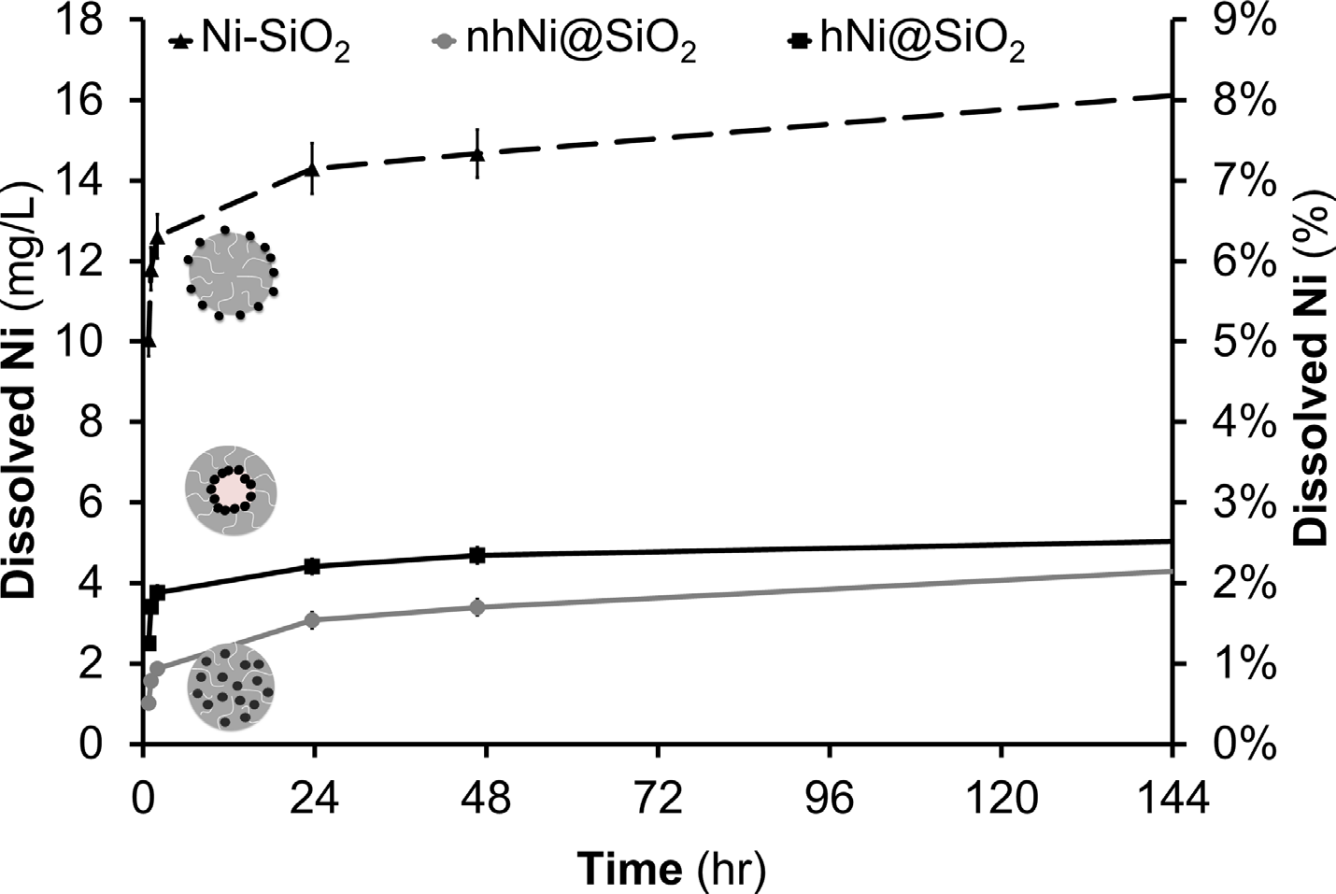
Nickel amount dissolved and percent dissolution of Ni-containing CEN in E3 medium at room temperature for 5 days with a starting concentration of 200 mg Ni/L. Ni-SiO_2_ had the highest Ni^2+^ dissolution.

Dissolution of metal ions from metal NPs is an important mechanism mediating the toxicity of metals [4, 16, 44, 59], including nickel [30, 31]. Therefore we asked if purely metal dissolution from the CENs into the medium correlated directly with toxicity to suggest a mechanistic link [60]. Nickel dissolution was determined by dispersing 200 mg Ni/L of CEN in E3 zebrafish embryo medium for five days. 200 mg Ni/L was chosen as it was the highest concentration used to analyze zebrafish larvae malformations and motility toxicity. This concentration also provides the maximum nickel ion dissolution expected over the course of the study. At specific time points, the CENs were separated using centrifugal ultrafiltration, and inductively coupled plasma atom emission spectroscopy (ICP-AES) was used to determine the amount of ionic nickel. For all materials, the rate of dissolution was initially rapid (one and four hour time point), but gradually slowed after the first 24 hours (Fig 2). Ni-SiO_2_ CENs showed the highest Ni ion dissolution (16.1 mg Ni/L) while hNi@SiO_2_ (5.04 mg Ni/L) and nhNi@SiO_2_ (4.3 mg Ni/L) CENs exhibited similar Ni ion concentrations at day five. However, the initial dissolution rate of hNi@SiO_2_ was almost double the nhNi@SiO_2_ dissolution rate. To furthermore assess potential differences in dissolution of the particles after uptake by the zebrafish embryos and larvae, i.e. to mimic the acidic conditions that the CEN will experience during endocytosis (i.e. in a lysosome), we also studied dissolution in a low pH environment (pH = 4.5). Dissolution was determined as described previously, but the E3 medium pH was adjusted to 4.5 by adding 0.1 M HCl dropwise over 15 minutes (~1–2 mL) until the monitored pH reached 4.5. While this cannot perfectly mimic the lysosome environment, these experiments yield insight into differences in dissolution due to pH differences alone, which is expected to be the main factor impacting dissolution behavior. As expected, all three CENs experienced significant enhanced nickel ion dissolution (over 75% total nickel) compared to the experiments at neutral pHs (S3 Fig) [61]. However, the qualitative behavior and relative ranking of the dissolution rates between the three CEN remained unchanged.

#### SiO2 dissolution

**Fig 3:**
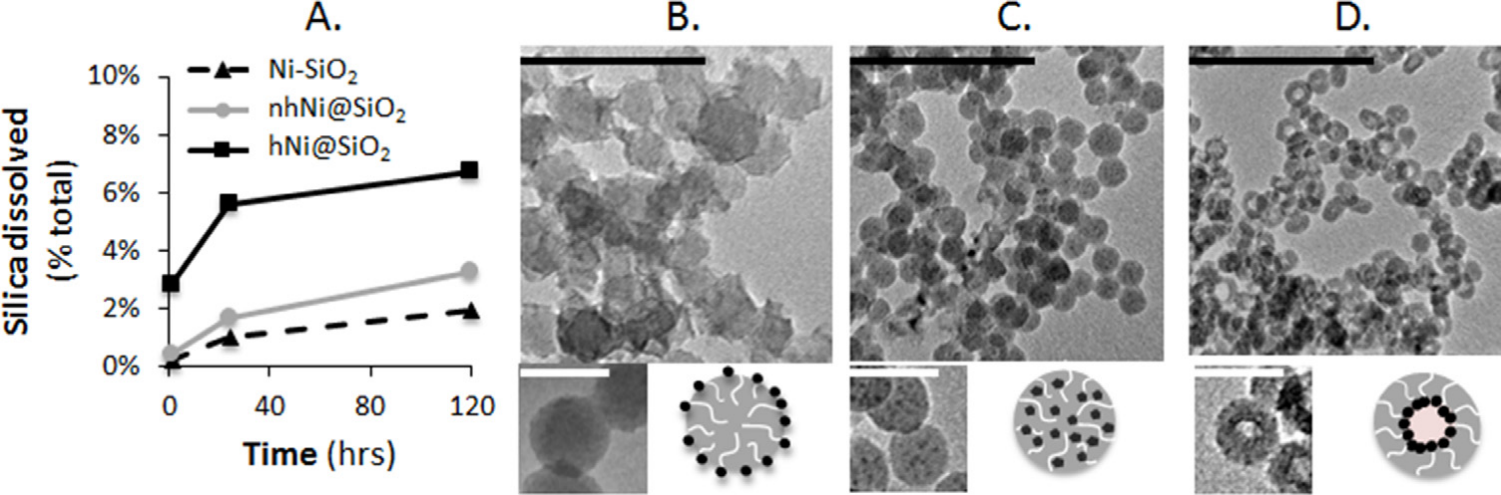
A. % total SiO_2_ dissolution from CENs dispersed in E3 medium for five days at room temperature with a starting concentration of 200 mg Ni/L. TEM images on day five of B. Ni-SiO_2_, C. nhNi@SiO_2_ and D. hNi@SiO_2_. Scale bars are 200 nm for larger images (black) and 50 nm for smaller pictures (white). All three CENs exhibited minimal silica dissolution.

Silica dissolution could compromise the nanomaterial stability in the aqueous media, alter the size and shape of the CEN, and ultimately cause the metal NPs to be released from the protective silica matrix into the medium or the zebrafish larvae, affecting the CEN’s toxicity. Therefore, silica dissolution was studied over five days by dispersing 200 mg Ni/L CENs in E3 media. TEM images were taken after the full five day exposure of the CENs, and silica dissolution was quantified at numerous time points using a colorimetric assay (Fig 3) [62]. hNi@SiO_2_ showed the highest dissolution (114 mg SiO_2_/L, ~6.7% total SiO_2_), followed by nhNi@SiO_2_ (55.9 mg SiO_2_/L, 3.3% total SiO_2_), and Ni-SiO_2_ (19.2 mg SiO_2_/L, 1.9% total SiO_2_). In agreement with the overall low dissolution, TEM shows only minor change in particle morphology. There is no discernible change in the decoration of the surface of Ni-SiO_2_ CENs with nickel NPs, and while some change in sphericity of the nhNi@SiO_2_ CEN and a slight thinning of the silica shell of the hNi@SiO_2_ CENs indicates some SiO_2_ dissolution, these changes remain minor and do not appear to affect the Ni NPs overall. An acidic environment, such as in the lysosome during nanomaterials uptake by the fish, further reduces silica dissolution to < 0.2% (S4 Fig) [63].

#### Ni/SiO2 CEN settling

**Fig 4:**
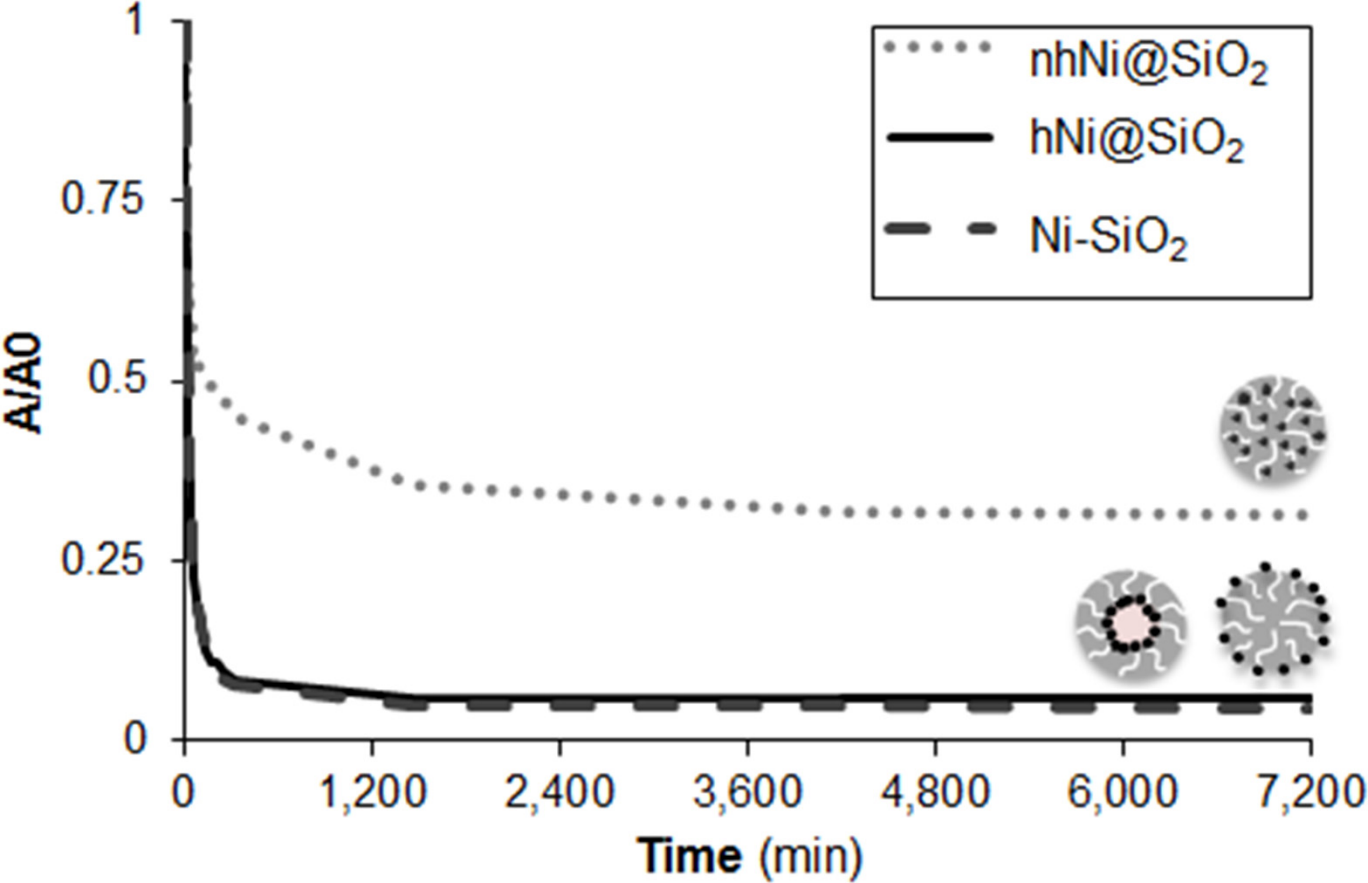
CEN Settling behavior measured via UV-vis spectroscopy over five days, shown as time-dependent absorbance (at λ- 287 cm^-1^, normalized to the initial absorbance A_0_ for each material). Initial concentration was 200 mg Ni/L. nhNi@SiO_2_ settled the least out of the three CENs.

Settling modifies the effective concentration in solution adjacent to the zebrafish, which at early developmental time points lie at the bottom of the wells [64]. 200 mg Ni/L CENs were dispersed in E3 medium and UV-visible spectroscopy was used to measure settling directly over five days (Fig 4). nhNi@SiO_2_ (68.5% decrease in absorbance over five days) settled significantly less than hNi@SiO_2_ and Ni-SiO_2_ (> 90% decrease in absorbance over five days).

### Toxicity of Ni/SiO_2_ CENs in zebrafish

#### Zebrafish survival

**Fig 5:**
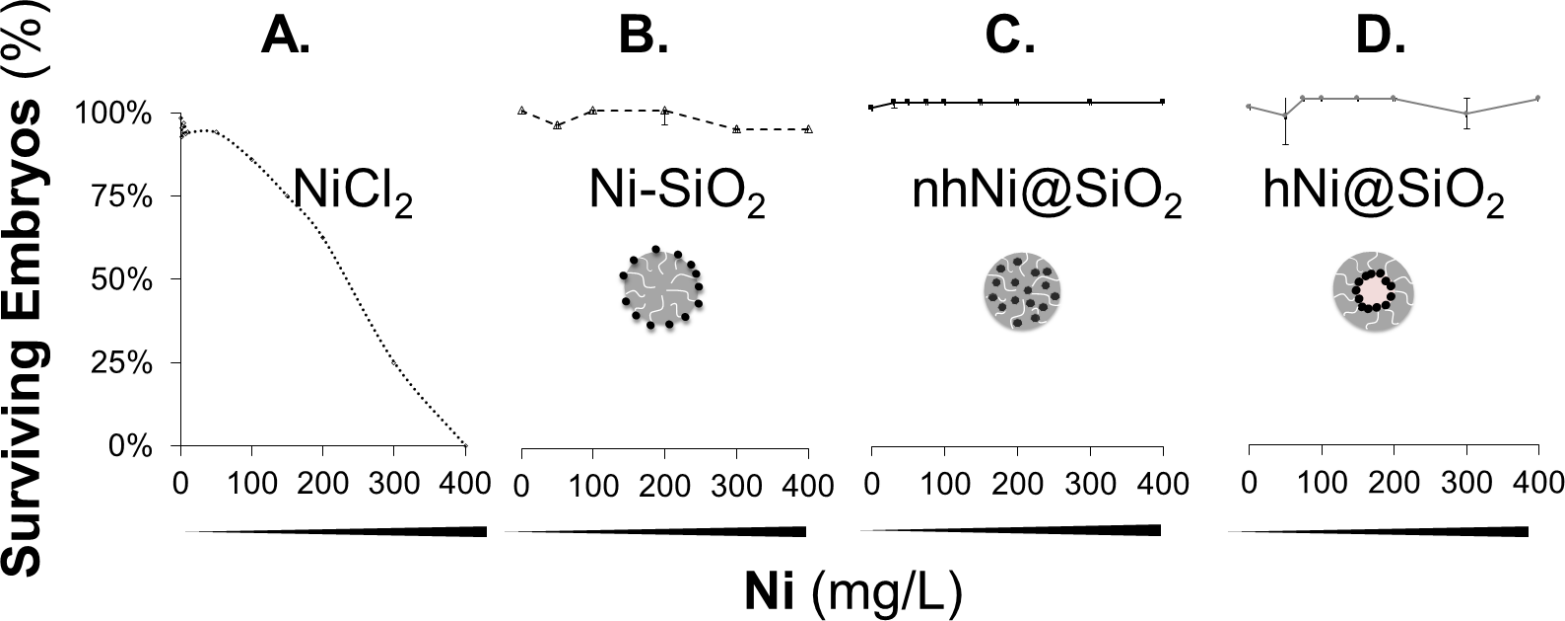
**Zebrafish larvae survival (%) after exposure to 5–400 mg Ni/L for A. NiCl**_**2**_ **B. Ni-SiO**_**2**_ **C. nhNi@SiO**_**2**_ **and D. hNi@SiO**_**2**_ **on 5dpf.** Error bars are error of sum squares (SSE). More than 95% zebrafish embryo survived following exposure to CENs at all concentrations tested.

To evaluate the toxicity of different Ni/SiO_2_ NP configurations, we exposed developing zebrafish embryos to 10 – 400mg Ni/L of CENs dispersed in E3 embryo medium, from 24 hours (Prim-5 stage) [21] to 5 days post-fertilization. It has been reported previously that the chorion can act as a barrier for uptake of metal nanoparticles [65, 66]. Consequently, in order to eliminate differences in chorionic penetration of nanoparticles as a variable from our studies, zebrafish embryos were dechorionated mechanically at 24 hours post-fertilization (hpf). Dechorionation is a standard method widely used in multiple labs worldwide for microscopy in live zebrafish. It is well-established that dechorionation does not itself adversely affect zebrafish development or health [67]. Survival (defined as a visible heart beat) was monitored daily until five days post-fertilization (dpf) (Fig 5). E3 medium containing NiCl_2_ was used as a positive control for toxicity related to dissolved Ni^2+^; E3 medium without additives was used as a negative control. Pure (nickel-free) silica NPs dispersed in E3 medium was used as a control to distinguish toxic effects of the silica support. In all experiments, we compared CEN, Ni^2+^, silica-only and E3-only exposures in identical dechorionated zebrafish embryos assigned to each group randomly, so that any differences between experimental groups are attributable unequivocally to the chemical exposure. 99% zebrafish embryos survived to 5dpf in E3 medium alone, or in E3 medium containing silica NPs at a concentration up to 2,700 mg SiO_2_/L. This corresponds to the amount of silica present at the highest concentration of CEN tested. As expected, NiCl_2_ was toxic in a concentration-dependent manner; the calculated LC_50_ for Ni^2+^ in this assay was 235 mg/L. In contrast, 95% embryos survived to 5dpf during exposure to all concentrations of CENs, including similar or higher total amounts of total (metallic + ionic) nickel to the NiCl_2_ group.

#### Zebrafish larvae malformations

**Fig 6:**
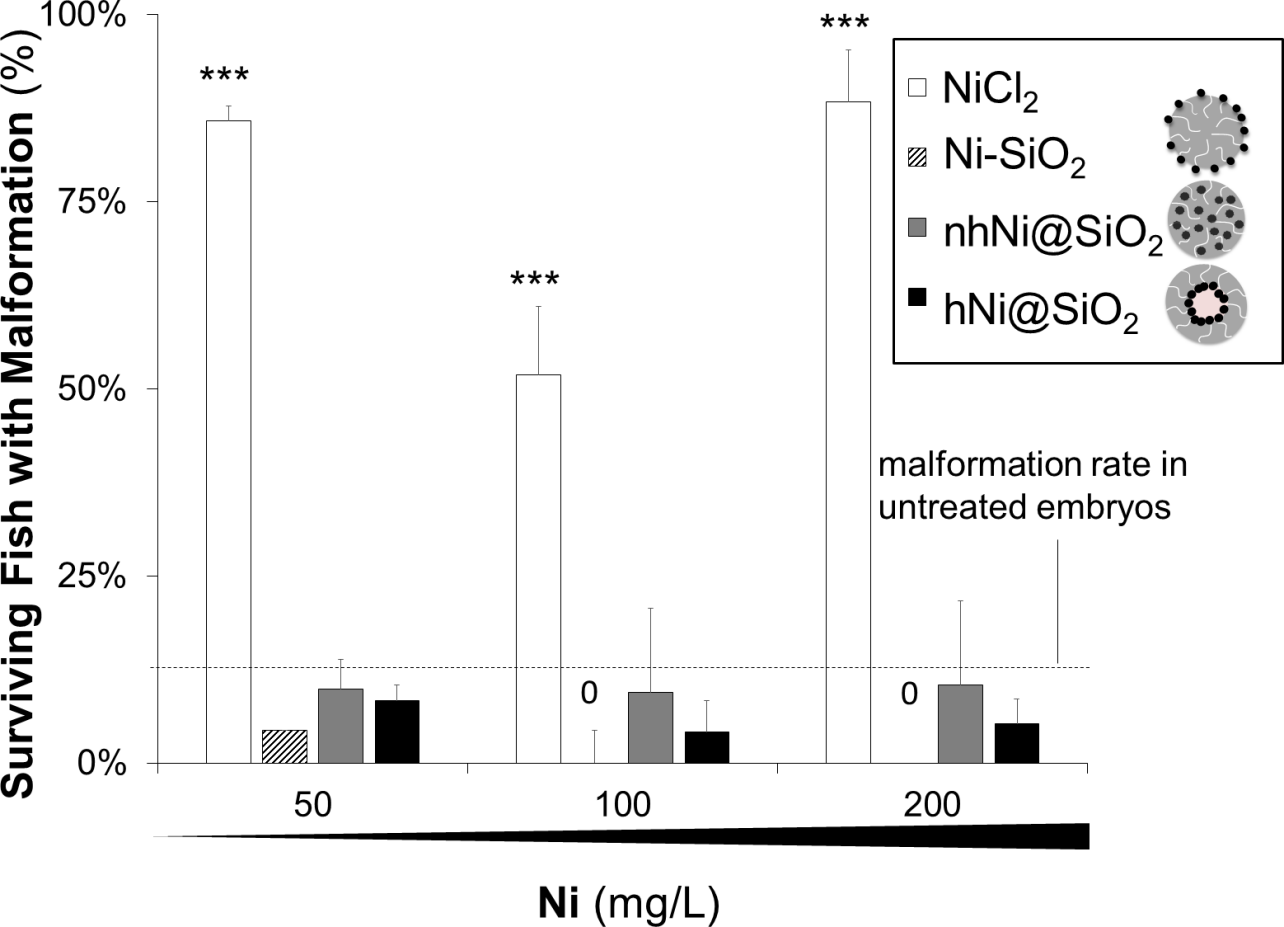
Malformations observed in surviving zebrafish larvae for NiCl_2_ and CENs on 5dpf. Malformation rate for unexposed control zebrafish larvae was 2.5% (indicated by the dashed line). Note that no malformations were observed for Ni-SiO_2_ at higher concentrations. ***p≥0.001 for one way ANOVA followed by Dunnett’s t-test, error bars are SSE. No significant development of malformations after exposure to Ni/SiO_2_ CENs.

Malformations were evaluated as an indicator of developmental toxicity. Metal NPs have previously been reported to cause a range of developmental defects in zebrafish larvae including abnormal spinal curvature, pericardial and abdominal edema [40, 41]. Zebrafish embryos were mechanically dechorionated at 24hpf and exposed to 10–200 mg Ni/L of CENs dispersed in E3 media. The embryos were monitored for the appearance of malformations over the next four days (Fig 6), including abnormal curvature of the spine and body (lordosis, kyphosis and scoliosis), pericardial and abdominal edema, and failure of swim bladder inflation (representative images and quantification of the frequency of different malformations is detailed in S5 Fig and S1 Table). One or more malformations were seen in a high proportion of zebrafish embryos exposed to NiCl_2_ (200 mg Ni/L: 88.3% ± 6.1%), whereas the malformation rate for embryos exposed to CENs containing a similar amount of Ni did not differ significantly from E3-only control (200 mg Ni/L nhNi@SiO_2_: 10.4% ± 11.2%; 200 mg Ni/L hNi@SiO_2_: 5.7% ± 6.1%; 200 mg Ni/L Ni-SiO_2_: 0.0% ± 0.0%; E3 medium only: 2.5% ± 2.0%; p<0.001 NiCl_2_ versus E3-only control, one-way ANOVA with Dunnett’s *post hoc* test).

#### Zebrafish larval motor function

The absence of detectable differences in toxicity between the CENs in survival or malformation assays prompted us to measure a functional endpoint that is sensitive to disruptions in neurological development. Previous reports indicate that nickel can act as a neurotoxin which would be expected to adversely affect motor function in developing zebrafish larvae [68–71]. Zebrafish larval motor behavior can be quantified in multiple larvae simultaneously, thereby allowing statistically robust determination of how toxicants alter motor physiology [72–74]. Three patterns of altered larvae locomotor behavior with increasing toxicant concentration have been reported previously: (i) a monotonic decrease in total larval displacement/time with increasing toxicant concentration [33, 43]; (ii) a monotonic increase in displacement/time with increasing toxicant concentration [75]; and (iii) a biphasic relationship in which locomotor activity first increases at lower concentrations and after reaching a maximum, decreases at higher concentrations [76].

**Fig 7:**
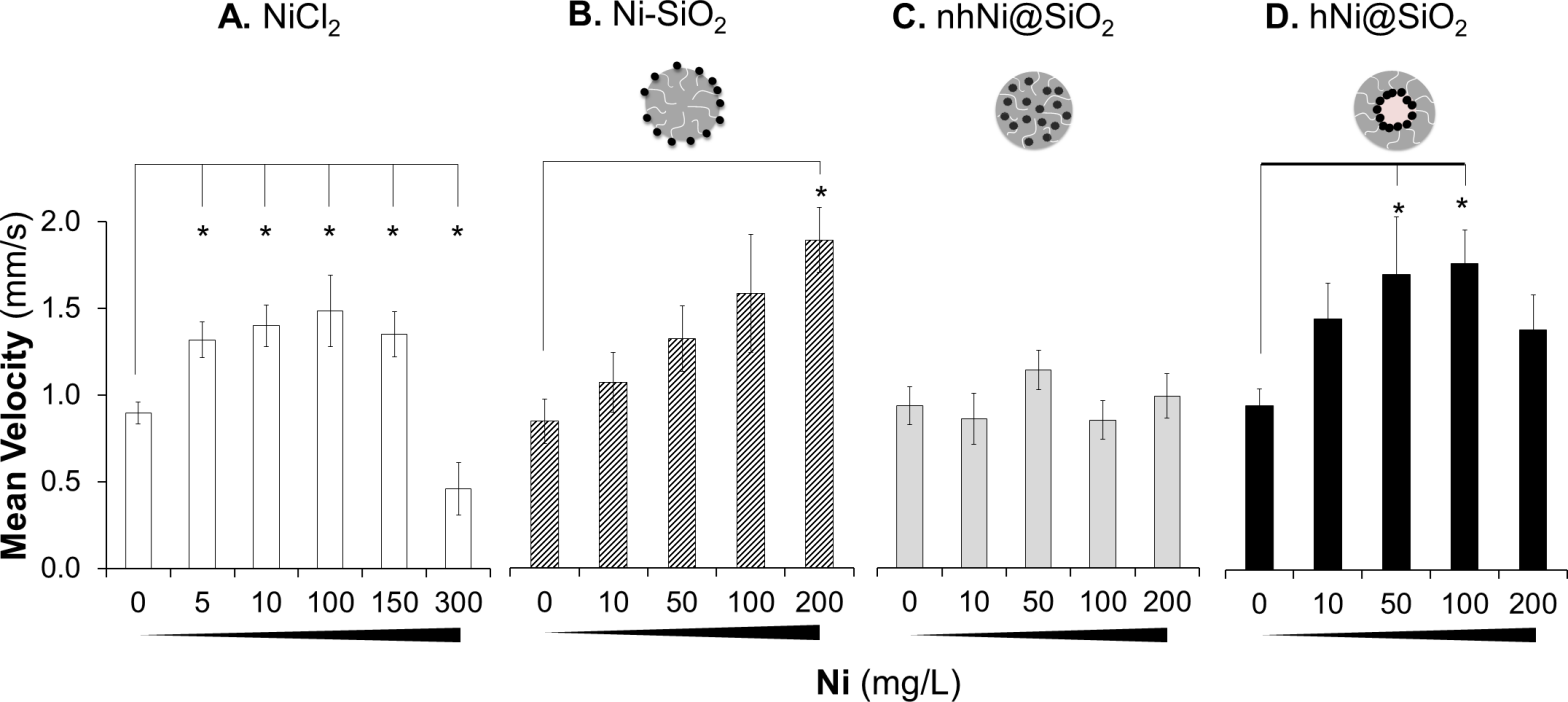
**Zebrafish larval mean velocity (mm/s) after exposure to 0–200 mg Ni/L A.) NiCl**_**2**_ **analogous salt and, B.) Ni-SiO**_**2**_**, C.) nhNi@SiO**_**2**_ **and D.) hNi@SiO**_**2**_ **CENs**. * indicates p≥0.05 for one-way ANOVA followed by Dunnett test, error bars SSE compared to E3 control. NiCl_2_, hNi@SiO_2_ and Ni-SiO_2_ caused a change in zebrafish larval mean velocity over the tested concentrations.

Embryos were mechanically dechorinated at 24hpf and exposed to Ni CENs for three days. Before the onset of exposure at the Prim-5 developmental stage (24hpf) [21], zebrafish embryos do not show spontaneous movement, which is first seen at Prim-30 (36hpf). By 5dpf, zebrafish larvae show rapid and regular spontaneous swimming behavior [74]. We therefore measured spontaneous propulsive movements at 5dpf, to evaluate the development of swimming behavior during CEN exposure. At 4dpf surviving zebrafish larvae were collected and transferred to E3 medium with no additives. All zebrafish larvae with morphological abnormalities (compared with normal healthy zebrafish larvae under light microscopy) were excluded from the motor assays. Consequently, the motor assays are informative about the development of locomotor function, rather than mechanical consequences or morphological malformation involving the body shape, trunk muscles or fins. Using our previously reported methods, we quantified zebrafish larval motor function at 5dpf in 96-well plates for one hour in bright white light (200 Lux brightness, 4900K color temperature) (Fig 7) [73, 74, 77], and determined their mean velocity (V_M_ = total displacement of the larval centroid over the course of the recording/ time period of observation). CEN-exposed animals were compared with controls derived from the same pool of dechorionated embryos, so that differences between the treatment groups were attributable unequivocally to chemical exposures rather than any baseline difference between animals. Compared with E3-only controls, pure nickel free SiO_2_ NPs, did not affect larval motility at any concentration tested (S6 Fig). Ni^2+^ salt provoked an increase in V_M_ at concentrations up to 100 mg Ni/L (Fig 7A). At higher concentrations (100–300 mg Ni/L) V_M_ declined below baseline measurements (detailed V_M_ ± SE and p-values for all materials and concentrations can be found in S1 File). Ni-SiO_2_ CEN caused a monotonic increase in V_M_ with increasing concentration through the concentration range measured (Fig 7B). Zebrafish embryos exposed to nhNi@SiO_2_ exhibited no change in larval V_M_ over the concentration range tested (Fig 7C). hNi@SiO_2_ provoked similar changes in motility to NiCl_2_ (Fig 7D); V_M_ increased from baseline up to a concentration of 100 mg Ni/L and then decreased again at higher concentrations. Together, these data show that developmental exposure to silica alone or nhNi@SiO_2_ did not provoke abnormalities in this assay. In contrast, hNi@SiO_2_ and NiCl_2_ provoked similar abnormalities to one another and Ni-SiO_2_ showed an intermediate phenotype. These data consequently allow us assign the following ranking to the toxicity of Ni CENs in the development of zebrafish larval motor function: hNi@SiO_2_ > Ni-SiO_2_ > nhNi@SiO_2._

#### Zebrafish nickel uptake

To determine how toxicity was correlated with nickel uptake, we measured tissue Ni concentrations in zebrafish embryos and larvae exposed to one of the three CENs or Ni^2+^. Zebrafish embryos were exposed to 50 mg Ni/L of either CEN or Ni^2+^ for 24–120 hours. After exposure, the zebrafish embryos and larvae were thoroughly washed five times and digested to allow measurement of tissue Ni content by ICP-AES. An ‘uptake efficiency’ was calculated after 48 or 96 hours of exposure as the measured nickel content per fish divided by the calculated available nickel ion concentration from NP dissolution (Fig 2). If only ionic Ni was taken up into the zebrafish larvae, the CEN uptake efficiency would be similar to the nickel salt.

**Fig 8:**
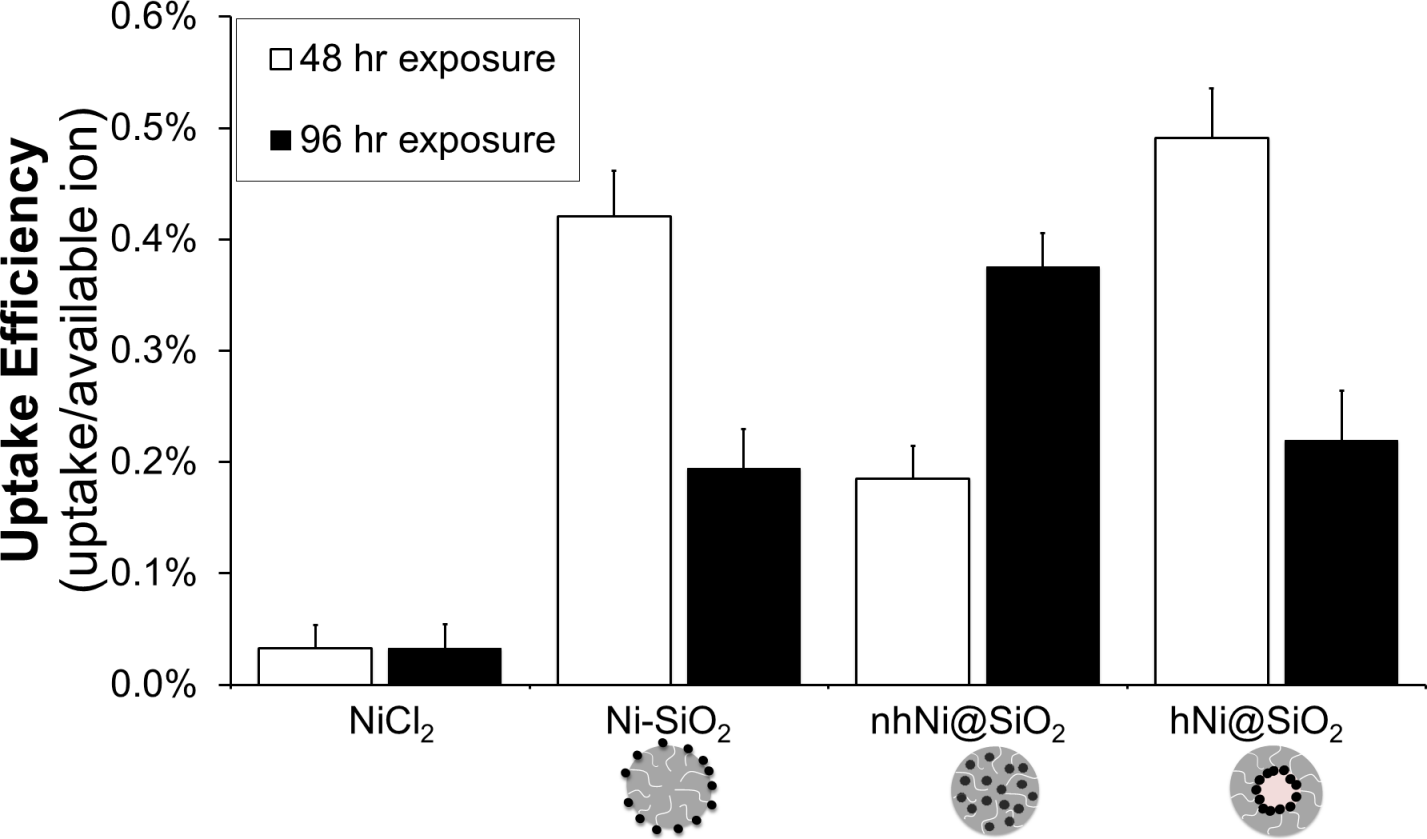
Zebrafish uptake efficiency of NiCl_2_ and CENs after 50 mg Ni/L exposure for 48 hours and 96 hrs.

**Fig 9:**
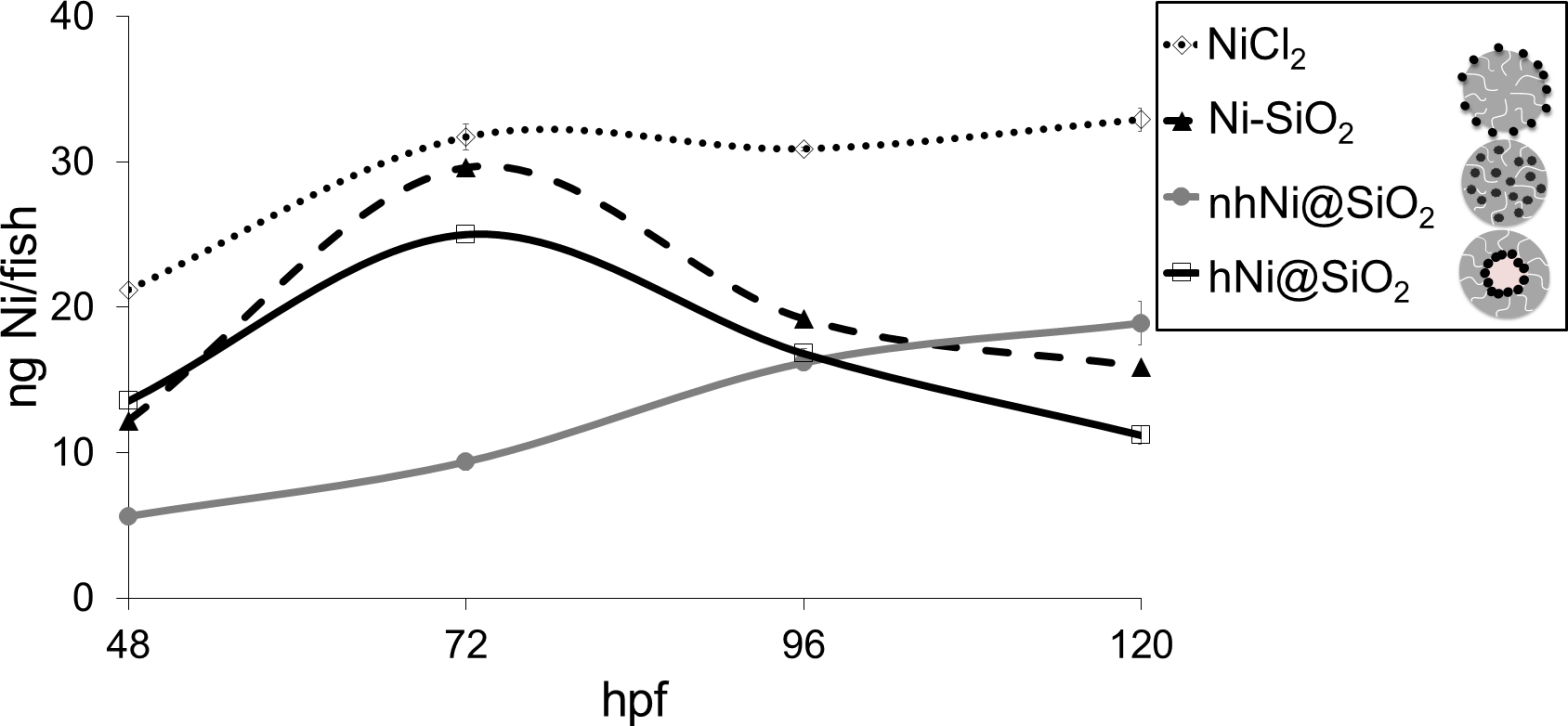
Zebrafish nickel uptake at 48, 72, 96, 120 hpf after exposure to 50 mg Ni/L NiCl_2_, Ni-SiO_2_, nhNi@SiO_2_ and hNi@SiO_2_.

The uptake efficiency was significantly higher for CENs than for Ni salt at both time points (Fig 8). This increased uptake indicates that the total Ni tissue content was derived from both uptake of ion and uptake of metal, indicating that CENs were likely taken up into the zebrafish larvae. Fig 9 shows the total zebrafish nickel content over the 96 hour exposure to the nickel materials. Overall, the zebrafish embryos exposed to NiCl_2_ had a higher internal nickel content than the zebrafish embryos exposed to Ni CENs. After exposure to NiCl_2_, the zebrafish Ni content increased over the first 24 hour exposure, but then reached a plateau and was steady over the next 72 hours. When comparing the three CENs, over the first 48 hour exposure, the zebrafish embryos exposed to Ni-SiO_2_ and hNi@SiO_2_ showed a higher nickel content than zebrafish embryos exposed to nhNi@SiO_2_. From 72–96 hour exposure, the total nickel content after exposure to Ni-SiO_2_ and hNi@SiO_2_ CENs decreased, pointing towards a nickel elimination mechanism [78]. In contrast, the internal nickel present after exposure to nhNi@SiO_2_ showed an increase from 48 to 96 hour exposure. The zebrafish embryos exposed to nhNi@SiO_2_ exhibited a continuous increase in internal nickel over the full 96 hour exposure and by 120 hpf had the highest nickel content out of all three CENs.

## Discussion

The present study aimed to utilize high-throughput zebrafish assays to study the toxicity of Ni/SiO_2_ CENs and understand structure-toxicity correlations that emerge from differing nanoconfigurations. Although they are simple and robust assays, we did not detect differences in survival or morphology between that were dependent on NP configuration, since all three CENs showed no difference from controls in either mortality or the presence of developmental malformations. In comparison, previous studies by Ispas et al. reported an LD50 of 200–300 mg Ni/L for 30–100 nm bare (i.e. unsupported) nickel NPs in similar 5-day zebrafish experiments [31]. The much smaller size of the Ni NPs in our study (~1–2 nm vs 30–100 nm) might be predicted to enhance toxicity as a consequence of the higher reactivity of smaller nanoparticles due to more under-coordinated, reactive surface sites, larger surface area, faster dissolution, etc. The absence of detectable mortality attributable to CEN in the present study strongly suggests that the silica embedding/deposition mitigates toxicity. Unfortunately, synthesizing 1–2 nm Ni nanoparticles free from residual synthesis chemicals—such as polymers [79], surfactants[80] and other chemicals [81] which would alter uptake and dissolution behavior and hence toxicity—without significant agglomeration and sintering of the particles is non-trivial. A direct comparison of the toxicity of bare Ni NPs as a function of size down into the sub-10 nm range has consequently not yet been achieved. However, our results provide initial support for the idea that embedding Ni NPs in a silica core significantly reduces their toxicity. This strategy potentially could be exploited in order to mitigate the toxicity of metal nanoparticles without impeding their functionality and thus has great potential to provide safer nanomaterials, for example, for use in chemical plants and in the fuel processing industry. These industries utilize metal NPs as catalysts and rely on supports to provide catalyst stability and thus could utilize these embedding strategies to reduce occupational hazard due to metal NP exposure.

In contrast to survival and morphology endpoints, the development of zebrafish larval motor function during CEN exposure provided an assay that distinguished the effects of the three different CENs. In this assay, hNi@SiO_2_ and Ni-SiO_2_ exhibited evidence of developmental toxicity while nhNi@SiO_2_ exposure throughout the concentration range tested did not alter development of motor function. The mechanism underlying the biphasic nature of the relationship between toxicants and the complex composite endpoint of whole organism motor function is not understood, though there has been at least one prior report of similar zebrafish motility behavior after exposure to alcohol [76]. However, it is clear that ‘toxicity’ is not a simple linear measurement in this assay and care must be taken to evaluate a sufficient concentration range of test compound to establish the nature of the relationship between concentration of toxicant and motor performance. Our study also suggests that it may be necessary to use multiple morphological and physiological endpoints in parallel to detect abnormalities provoked by novel putative toxicants, since evaluation of only morphology or survival in this study would have missed potentially significant abnormalities triggered by CENs.

Our physicochemical characterization of the CENs allowed us to identify properties that affect the toxicity of nanomaterials. First, the observed differences in nickel dissolution behavior can be traced back to the structural differences between the three CENs: For Ni-SiO_2_, the external nickel NPs are directly exposed to the bulk medium and are therefore subject to fast dissolution. In contrast, the nickel NPs in hNi@SiO_2_ (which can be considered an “inverted” configuration of the Ni-SiO_2_) are exposed only to the solution inside the cavity of the silica shell. The small liquid volume contained in the central cavity likely develops a rapid increase in Ni^2+^ concentration from Ni NP dissolution, which slows further dissolution. Exchange of the liquid volume in the central cavity with the bulk solution surrounding the CEN through the microporous silica walls assures continued, but slow, dissolution. Finally, the Ni NPs in nhNi@SiO_2_ are tightly embedded into the porous silica matrix, reducing not only the liquid volume into which Ni dissolves but also the surface area of the Ni NPs directly exposed to the solution. Furthermore, compared with hNi@SiO_2_, the silica pores of nhNi@SiO_2_ are significantly longer as they extend throughout the silica particle, rather than just through the walls. These factors likely combine to retard dissolution by reducing the effective solvent volume, exposed NP surface area and rate of solvent exchange between pores and the surrounding media. However, if toxicity were entirely dependent on Ni^2+^ dissolution into the media, these results suggest that Ni-SiO_2_ should be significantly more toxic than either hNi@SiO_2_ or nhNi@SiO_2_, yet we found hNi@SiO_2_ to be more toxic than Ni-SiO_2_. Furthermore, the maximum Ni^2+^ concentration that resulted from dissolution of Ni from the CENs into the medium was 16 mg Ni/L–the corresponding concentration of NiCl_2_ did not provoke abnormalities, and so toxicity cannot be explained solely by ion dissolution from the CENs into the media.

Silica dissolution was found to be minor for all three CENs. While silica dissolution may affect toxicity for longer exposures times (past five days when the silica matrix may show more substantial dissolution), silica dissolution does not pose a toxicity concern during these subacute exposures as the silica structures prove to be sufficiently robust.

In contrast, the developmental toxicity associated with hNi@SiO_2_ and Ni-SiO_2_ correlated with their settling behavior. Rapid settling is predicted to result in a higher effective CEN concentration towards the bottom of the well. Since zebrafish embryos lie at the bottom of the well until they become motile, an elevated concentration of CENs at the bottom of a well could potentially lead to higher CEN uptake in the zebrafish during early development [19]. After development of spontaneous motility after 36hpf, larval zebrafish start to spend less time at the bottom of the well. The more toxic CENs we tested (hNi@SiO_2_ and Ni-SiO_2_) showed rapid settling, consistent with the idea that enhanced exposure earlier during development (24 – 48hpf) accounted for the detected abnormalities of motor function at 5dpf. Possible explanations for sensitivity of motor function to disruption during early development might include blood-brain barrier formation that might exclude metal ions from the CNS at later time points or disruption of the formation of circuits essential for motor function during a critical window early in development. Regardless, alterations in effective exposure caused by settling are an important additional variable in the evaluation of CENs that settle and should be taken into account in future studies.

Interestingly, hNi@SiO_2_ proved to be more toxic than the other two CENs, even though Ni-SiO_2_ showed faster dissolution in E3 medium and caused zebrafish total nickel concentration to be higher. Our assay for measuring zebrafish Ni content does not differentiate ionic from metallic Ni. We predict that Ni dissolution inside a zebrafish will differ significantly from the external solution because the pH differs and there is a high concentration of proteins within the zebrafish that could form a corona around the CENs. These corona would be expected to limit dissolution of Ni from Ni-SiO_2_ by coating the external nickel NPs, whereas the nickel NPs embedded inside hNi@SiO_2_ might be protected by the silica matrix from protein capping, resulting in enhanced dissolution inside the animal. If toxicity were dependent on Ni^2+^ inside the zebrafish, this is a possible mechanism by which hNi@SiO_2_ shows enhanced toxicity compared with the other structures we studied.

## Conclusion

Our results demonstrate that zebrafish embryos provide a useful screening model for evaluating CEN toxicity during vertebrate development by combining established (zebrafish larval survival and malformations) and novel (zebrafish larval locomotor function) methods for detecting phenotypes. Based on these assays, we found that Ni/SiO_2_ CENs were significantly less toxic than the corresponding ionic metal and might offer a potential path towards mitigating NP toxicity (formal proof of the latter awaits the development of suitable techniques for the synthesis of size-controlled nickel NPs smaller than 10nm without capping agents). Our results highlight the importance of conducting a thorough physicochemical characterization of nanomaterials in the biological system of interest as an inherent part of the toxicity assessment. While ex-situ characterization (i.e., TEM, XRD, BET surface area, etc.) is necessary to evaluate baseline CEN properties, it is not sufficient to elucidate size- and structure-dependent effects that occur in the test media, such as settling, agglomeration, and dissolution. Based on these characterizations of CENs in biological media, our results suggest that modification of the effective exposure might be more important for determining NP toxicity than “nano-chemistry” effects. Overall, we propose that CENs may offer a relatively straightforward stepping stone towards the rational design of safer nanomaterials.

## Methods

### CEN Synthesis

Ni/SiO_2_ CENs were made using a one-pot, multi-step reverse microemulsion synthesis previously developed in our laboratory. The three nanomaterials utilized variations of the same synthesis protocol, assuring close structure similarity. Hollow Ni@SiO_2_ (hNi@SiO_2_) materials were synthesized using a one-pot reverse microemulsion synthesis previously developed in our laboratory. First, 50 mL of cyclohexane (≥99%) and 10.5 g surfactant Brij 58 (≥99% polyethylene glycol hexadecyl ether, Mn ~1124, Sigma-Aldrich) were refluxed at 50°C until the surfactant was fully dissolved. 1.5 mL of 1 M Ni(NO_3_)_2_^.^6H_2_O (99.999%) was then added drop-wise, followed by 1.5 mL hydrazine hydrate (Sigma-Aldrich) to form a nickel hydrazine complex. Next, 5 g of tetraethylorthosilicate (TEOS, ≥99%) was added, followed by 3 mL of ammonium hydroxide (30%). After 2 hours of aging for silica growth, particles were precipitated with 2-propanol, collected via centrifugation, washed three times with 2-propanol, and dried in air. The crushed powder was then calcined in a Thermolyne 79300 tube furnace for 2 hours at 500°C in air.

Non-hollow Ni@SiO_2_ (nhNi@SiO_2_) CENs were made by a simple modification of the hNi@SiO_2_ synthesis method, omitting the hydrazine addition step. Absence of the micelle-stabilizing Ni-hydrazine complex results in the formation of a solid (but porous) silica particle with embedded Ni NPs throughout the silica matrix. The precipitated material was dried and calcined as described above. To remove external nickel from the materials, calcined particles were reduced and etched in nitric acid by dispersing 0.20 g of material in aqueous nitric acid (35 vol%) for 30 min. The etched materials were washed twice in water to neutral pH, dried, and calcined.

For surface-deposited Ni-SiO_2_, nickel-free spherical silica spheres were first synthesized using the microemulsion nhNi@SiO_2_ procedure above but replacing aqueous Ni salt with 1.5 mL deionized (DI) water. Following calcination of the Ni-free silica, a deposition-precipitation method, modified from Deng et al. [82], was used to deposit very small and near-monodisperse nickel NPs on the surface of the calcined silica spheres. 0.6 g of silica NPs were dispersed in 15 mL of DI water by sonication, Ni salt solution was added (0.55 g NiCl_2_ in 10 mL DI water), and the mixture was again sonicated for 20 min. Ammonium hydroxide (30%) was then added drop-wise (~5 mL, 52 drops, slowly over 20 min) until the pH of the solution was ~9.5. The resulting material was mixed for 20 min, centrifuged, dried, calcined at 300°C in air, rinsed twice in DI water, dried, and calcined again at 300°C in air.

### CEN Characterization

CEN size and morphology were characterized with transition electron microscopy (TEM, JEOL-2000FX electron microscope). Particle measurements of TEM images were done using ImageJ software (http://rsb.info.nih.gov/ij/). Scanning Electron Microscopy (SEM) equipped with EDX was used at beam voltage of 15kV to determine elemental composition. Surface area and porosity were determined by Brunauer Emmett Teller (BET) analysis using a Micromeritics ASAP 2020 surface area and porosity analyzer. Pre-treatment consisted of 2–3 hr degassing at 200°C under vacuum. Typically, a 6-point BET analysis was used for total surface area measurement and an 84-point N_2_ Barrett-Joyner-Halenda (BJH) analysis with Halsey thickness curve correction and Kruk-Jaroniec-Sayari correction for pore size and volume determination.

CEN dispersions were tested by dynamic light scattering (DLS, Zetasizer Series Nano-ZS) to estimate the hydrodynamic diameter of the CEN in E3 medium (49 mM NaCl, 1.6 mM KCl, 3.3 mM CaCl_2_, 3.3 mM MgSO_4_, pH 7.4). E3 medium was chosen as it is the zebrafish medium used for the toxicity studies. The samples were first dispersed by sonication and then ~1 mL of dispersed solution was added to a cuvette. The refractive index of silica was used for all measurements. CEN Settling was measured by UV-Visible spectroscopy (Beckman Coulter DU720). Path length was 1 cm and wavelength was 287 nm. CENs were dispersed in E3 medium and deposited in a cuvette to a liquid height of 1.27 cm. Dissolved trace metal ion concentration was measured under radial detection by inductively coupled plasma atomic emission spectroscopy (ICP-AES, Thermo Electron Corporation iCAP6500 Duo Series ICP-OES Spectrometer). Standards were formulated from a stock standard solution (Fischer Scientific) with 3 wt% HNO_3_ in deionized water to generate a standard curve. The degree of nickel ion dissolution from the CENs in E3 medium was determined at specific time points. 10 mL dispersions (200 mg Ni/L) were prepared in E3 media. CENs were removed from the dispersions by centrifugation followed by filtration (Amicon 10,000 molecular weight cut-off filters, ~3.1 nm). HNO_3_ (Sigma, 70%) was added dropwise to the filtrate to a concentration of 3 wt%. Dissolved silica concentration was measured using the ASTM D859-00 standard test method for silica in water [62]. Briefly, 10 mL dispersions (200 mg Ni/L) were prepared in E3 media. At specific time points the CENs were removed by centrifugation followed by filtration. 0.2 mL of HCl (1:1 water:acid) and 0.4 mL of ammonium molybdate (75 g/L, Sigma-Aldrich) was added in succession to the collected sample. After five minutes, 0.3 mL of oxalic acid (100 g/L, Sigma-Aldrich) was added. 0.4 mL of amino-naphthol-sulfonic acid (0.5 g 1-amino-2-naphthol-4-sulfonic acid + 150mL DI water + 1 g Na_2_SO_3_ + 30 g NaHSO_3_) was added after 1 minute. After ten minutes the absorbance was read at 815 nm using UV-Visible spectroscopy. A blank was prepared using E3 medium without CENs.

### Zebrafish

Zebrafish studies were carried out in compliance with all federal and local regulations, in accordance with NIH guidelines for animal care and use and with full approval from the University of Pittsburgh Institutional Animal Care and Use Committee. Embryos for experiments were generated by crossing healthy adult WT strain AB zebrafish of 4–12 months of age. The afternoon before mating, zebrafish were placed in breeding tanks with false bottoms, and dividers to separate males and females. On the morning of mating the divider was removed at 08:00 when the zebrafish facility lights are illuminated to allow breeding. Timed embryos were collected within the first hour of breeding. The embryos were washed in system water and then E3 buffer and transferred to 10cm plates containing E3 buffer with methylene blue (0.0001% w/v). A maximum of 30 embryos were housed in each dish. Plates were kept at 28.5°C in an incubator with white light illumination (color temperature 4900K; brightness 200 Lux) on a light-dark cycle (14 hours light:10 hours dark, light starts at 08:00). At 24 hours post-fertilization (hpf) embryos that showed developmentally appropriate morphology, Prim-5 as defined by Kimmel et. al. [21], and a visible heart beat were mechanically dechorionated and transferred to fresh E3 without methylene blue at 28.5°C. After two further rinses in E3 without methylene blue, animals were randomly split into separate experimental groups and one healthy embryo was transferred to each well of a 24-well plate. Dried CEN powder was weighed, added to E3 media, and sonicated 20 min. Serial dilutions were then prepared volumetrically. The solutions were re-dispersed by sonication for 1–2 minutes immediately prior to addition to the well. The bulk of the E3 buffer was removed from the well prior to adding the nanoparticle suspension, so that any dilution of the nanoparticle from E3 carryover was minimal quantitatively insignificant. In accordance with standard zebrafish rearing procedure no external source of nutrients was added during the 4 days exposure while zebrafish naturally depleted their yolk [83]. Zebrafish embryos were mechanically dechorionated and incubated for four days at 28.5°C and analyzed visually each day for survival and developmental malformations. Solutions were not changed over the course of the experiment to eliminate a buildup of NP concentration during the multi-day exposures. The excess volume of buffer in each well was adequate to ensure that the control groups developed normal morphology and motor function. 12–18 embryos were tested at each concentration of toxicant per experiment.

Zebrafish motor function was analyzed by exposing embryos to CENs at 24hpf as detailed above; surviving larvae were collected on day 4, zebrafish larvae displaying morphological abnormalities were excluded from the motor assays. The zebrafish larvae were washed three times in E3 medium to remove residual particles by being gently transferred in E3 using a Pasteur pipette. The washed zebrafish were then transferred to a 96-well plate. Motor function was then analyzed as described in detail in our previous work [73, 77]. Briefly, a video stream of zebrafish moving in the wells of a 96-well plate was captured at 2 frames/s. Video recordings were analyzed using the open source *LSRtrack* and *LSRanalyze* MATLAB scripts that we reported and extensively validated previously [73, 74, 77]. All video recordings were taken at the same time of the day (between 2–5 pm).

Nickel uptake was determined by collecting surviving embryos and larvae upon termination of the experiment to measure cumulative metal uptake. Briefly, larvae were transferred to clean 12-well plates in groupings by dose, rinsed 5 times in fresh E3 medium to rinse residual external particles from surface of the zebrafish, euthanized using 10% sodium hypochlorite and rinsed 5 times with E3 media. Residual medium was then removed and zebrafish were dried in air. A dissolving procedure by Borgmann et al. [84] was used beginning with addition of nitric acid (12.5 μL/fish, 70%, Fischer Scientific) followed by at least one week digestion followed by addition of hydrogen peroxide (10 μL/fish, 30%, JTBaker) and 24 hours digestion. Resulting solutions were diluted up to 8 mL and the Ni concentration was measured via ICP-AES. The number of rinse cycles was determined by repeating washes until the nickel uptake measurement became independent of the number of washes. Furthermore, an independent experiment was conducted with fluorescent CENs, confirming the absence of detectable CENs attached to the zebrafish larvae exterior after five washes.

### Statistics

Data for each of the assays were parametrically distributed. To compare the effects of multiple different concentrations of each nanomaterial with embryo buffer in the same experiment, we employed one-way ANOVA followed by Dunnett’s test [85]. * indicates p≤0.05 and *** indicates p≤0.001. Three independent experiments were conducted for each zebrafish endpoint with 12–18 fish per condition.

## Supporting Information

S1 Fig BET surface area and porosity for SiO_2_, Ni-SiO_2_, nhNi@SiO_2_, ^hNi@SiO2.^ (TIF)

S2 Fig X-ray diffraction patterns of hNi@SiO_2_, nhNi@SiO_2_, and Ni-SiO_2_. Square = Ni (04–0850), circle = NiO (78–0643), triangle = NiSiO_3_ (43–00664).
(TIF)

S3 Fig **A. Quantitative 200 mg Ni/L CEN nickel ion dissolution in E3 with a pH = 4.5 over 120 hours**. Representative TEM images B. Ni-SiO_2_ C. nhNi@SiO_2_ and D. Ni-SiO_2_.
(TIF)

S4 Fig Relative dissolution of silica (shown as percentage of total initial silica amount) for 200 mg Ni/L CENs dispersed in E3 media with pH adjusted to 4.5 to mimic the lysosome environment. (TIF)

S5 Fig NiCl_2_-induced developmental malformations. Examples are shown of zebrafish larval malformations present at 5 dpf, following exposure to NiCl_2_ (50 mg Ni/L) for 4 days. A. Control, healthy zebrafish with normal morphology, including swim bladder (SB) formation. B. Zebrafish with abnormal spinal curvature including kyphotic (Ky) and lordotic (Lo) deformities. C. Zebrafish with more prominent lordosis (Lo) and pericardial edema (PE) D. Zebrafish with severe lordosis (Lo), pericardial edema (PE) abdominal edema (AE), tissue necrosis (TN), abnormal fin morphology (FM) and small eyes (E). Note panels B-D show failure of swim bladder formation; C and D also show shortened body length. C also shows lateral spinal curvature (scoliosis) seen as the caudal extremity of the body being out of the plane of focus of the micrograph.
(TIF)

S6 Fig Zebrafish mean velocity (mm/s) after exposure to 0–2700 mg SiO_2_/L. * indicates p≥0.05 for one-way ANOVA followed by Dunnett test, error bars SSE.
(TIF)

S1 File Detailed zebrafish mean velocity. (PDF)

S1 Table Frequency of malformations following developmental NiCl_2_ exposure. The table shows the frequency (mean ± standard deviation) of abnormal spinal curvature, abdominal edema and pericardial edema after exposure to 50, 100 and 200 mg Ni/L NiCl_2_ and CENs. Each individual zebrafish larva could show more than one malformation and all were recorded. The malformation rate was normalized to the number of surviving zebrafish. Note: after exposure to 100 and 200 mg Ni/L Ni-SiO_2_ zebrafish developed no malformations.
(PDF)
