# Supplementary figures and images for: The Developmental Toxicity of Complex Silica-Embedded Nickel Nanoparticles Is Determined by Their Physicochemical Properties
Source: PLoS One. 2016 Mar 31;11(3):e0152010. doi: 10.1371/journal.pone.0152010 (PMC4816503; doi:10.1371/journal.pone.0152010)

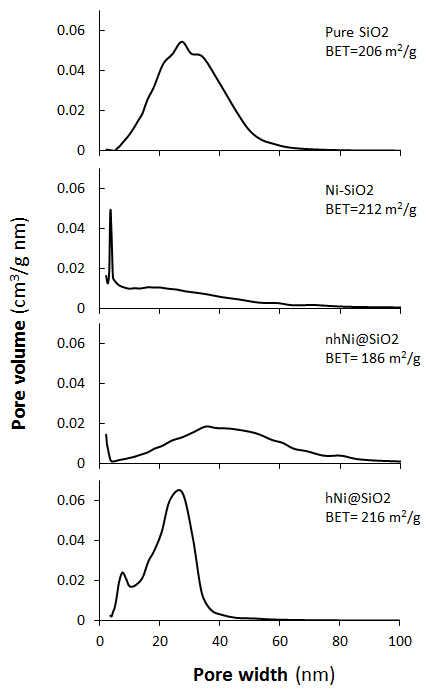

Supplement: S1 Fig — (TIF) [file pone.0152010.s001.tif]

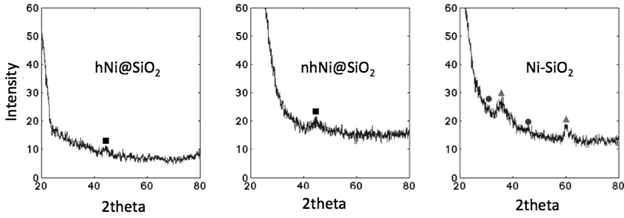

Supplement: S2 Fig — Square = Ni (04–0850), circle = NiO (78–0643), triangle = NiSiO3 (43–00664). (TIF) [file pone.0152010.s002.tif]

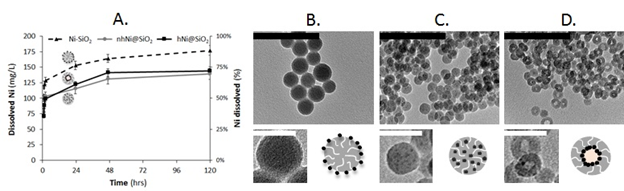

Supplement: S3 Fig — A. Quantitative 200 mg Ni/L CEN nickel ion dissolution in E3 with a pH = 4.5 over 120 hours. Representative TEM images B. Ni-SiO2 C. nhNi@SiO2 and D. Ni-SiO2. (TIF) [file pone.0152010.s003.tif]

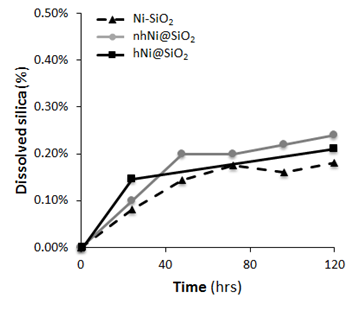

Supplement: S4 Fig — (TIF) [file pone.0152010.s004.tif]

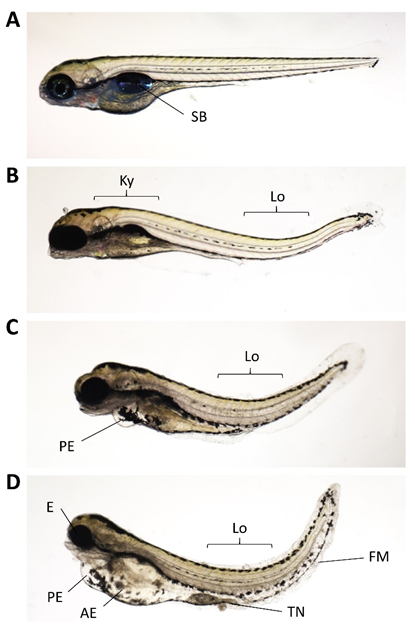

Supplement: S5 Fig — Examples are shown of zebrafish larval malformations present at 5 dpf, following exposure to NiCl2 (50 mg Ni/L) for 4 days. A. Control, healthy zebrafish with normal morphology, including swim bladder (SB) formation. B. Zebrafish with abnormal spinal curvature including kyphotic (Ky) and lordotic (Lo) deformities. C. Zebrafish with more prominent lordosis (Lo) and pericardial edema (PE) D. Zebrafish with severe lordosis (Lo), pericardial edema (PE) abdominal edema (AE), tissue necrosis (TN), abnormal fin morphology (FM) and small eyes (E). Note panels B-D show failure of swim bladder formation; C and D also show shortened body length. C also shows lateral spinal curvature (scoliosis) seen as the caudal extremity of the body being out of the plane of focus of the micrograph. (TIF) [file pone.0152010.s005.tif]

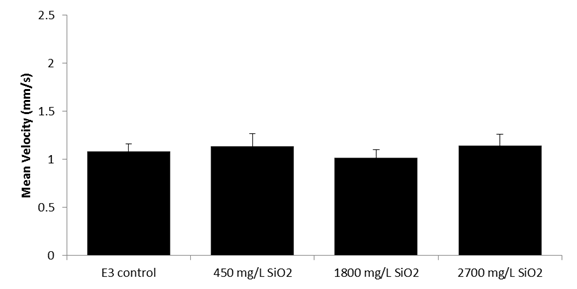

Supplement: S6 Fig — * indicates p≥0.05 for one-way ANOVA followed by Dunnett test, error bars SSE. (TIF) [file pone.0152010.s006.tif]
