# Supplementary material for: The Developmental Toxicity of Complex Silica-Embedded Nickel Nanoparticles Is Determined by Their Physicochemical Properties
Source: PLoS One. 2016 Mar 31;11(3):e0152010. doi: 10.1371/journal.pone.0152010 (PMC4816503; doi:10.1371/journal.pone.0152010)
Supplement: S1 File — (PDF) [file pone.0152010.s007.pdf]

S8 Text: Detailed Mean Velocity

$NiCl_2$ salt: A non-monotonic, inverted, u-shaped concentration/mean velocity relationship was observed in (Fig 7A). Up to 100 mg/L, mean velocity increased with $NiCl_2$ concentration: 0 mg Ni/L: 0.895 ± 0.06 mm/s; 5 mg Ni/L: 1.32 ± 0.10 mm/s p=0.004; 10 mg Ni/L: 1.33 ± 0.09 mm/s p=0.006; 100 mg Ni/L: 1.49 ± 0.20 mm/s p=0.003; 150 mg Ni/L: 1.35 ± 0.13 mm/s p=0.032. At higher concentrations, $NiCl_2$ caused a decline in mean velocity below baseline measurements: 300 mg Ni/L: 0.46 ± 0.15 mm/s p=0.020; p is one ANOVA followed by Dunnett's test.

Ni-SiO_2: A monotonic increase in mean velocity was observed over the entire concentration range (Fig 7B): 0 mg Ni/L: 0.851 ± 0.12 mm/s; 10 mg Ni/L: 1.08 ± 0.17 mm/s p=0.801; 50 mg Ni/L: 1.32 ± 0.19 mm/s p=0.122; 100 mg Ni/L: 1.58 ± 0.34 mm/s p=0.081; 200 mg Ni/L: 1.90 ± 0.18 mm/s p=0.001; p is one-way ANOVA followed by Dunnett's test.

nhNi@SiO_2: No effect on mean velocity was observed over the entire concentration range tested (Fig 7C): 0 mg Ni/L: 0.937 ± 0.10 mm/s; 10 mg Ni/L: 0.860 ± 0.14 mm/s; 50 mg Ni/L: 1.14 ± 0.11 mm/s; 100 mg Ni/L: 0.85 ± 0.11 mm/s; 200 mg Ni/L: 0.99 ± 0.12 mm/s, p= 0.83; p is one-way ANOVA.

hNi@SiO_2: A mean velocity increase was observed up to a concentration of 100 mg/L (Fig 7D): 0 mg Ni/L: 0.950 ± 0.09 mm/s; 10 mg Ni/L: 1.45 ± 0.20 mm/s p=0.261; 50 mg Ni/L: 1.71 ± 0.31 mm/s p=0.032, 100 mg Ni/L: 1.77 ± 0.19 mm/s, p=0.042. At higher concentrations, mean velocity decreased towards baseline values: 200 mg Ni/L: 1.39 ± 0.20 mm/s p=0.264; p is one-way ANOVA followed by Dunnett's test.
