# Supplementary material for: The Developmental Toxicity of Complex Silica-Embedded Nickel Nanoparticles Is Determined by Their Physicochemical Properties
Source: PLoS One. 2016 Mar 31;11(3):e0152010. doi: 10.1371/journal.pone.0152010 (PMC4816503; doi:10.1371/journal.pone.0152010)
Supplement: S1 Table — The table shows the frequency (mean ± standard deviation) of abnormal spinal curvature, abdominal edema and pericardial edema after exposure to 50, 100 and 200 mg Ni/L NiCl2 and CENs. Each individual zebrafish larva could show more than one malformation and all were recorded. The malformation rate was normalized to the number of surviving zebrafish. Note: after exposure to 100 and 200 mg Ni/L Ni-SiO2 zebrafish developed no malformations. (PDF) [file pone.0152010.s008.pdf]

S6 Table. Frequency of malformations following developmental $NiCl_2$ exposure. The table shows the frequency (mean ± standard deviation) of abnormal spinal curvature, abdominal edema and pericardial edema after exposure to 50, 100 and 200 mg Ni/L $NiCl_2$ and CENs. Each individual zebrafish larva could show more than one malformation and all were recorded. The malformation rate was normalized to the number of surviving zebrafish. Note: after exposure to 100 and 200 mg Ni/L Ni-SiO_2 zebrafish developed no malformations.

Material/Concentration		Abnormal spinal curvature	Abdominal edema (AE)	Pericardial edema (PE)
Control	0 mg Ni/L	1.2± 1.1%	0 ± 0.0%	0± 0.0%
$NiCl_2$	50 mg Ni/L	58.3± 3.1 %	13.7± 4.7%	48.0± 4.0%
	100 mg Ni/L	37.3± 2.5%	16.6± 2.6%	33.7± 5.1%
	200 mg Ni/L	66.3± 3.9%	35.3± 5.5%	62.7± 2.9%
Ni-SiO_2	50 mg Ni/L	2.8± 1.5%	0.3± 0.1%	0.3± 0.1%
	100 mg Ni/L	0± 0.1%	0± 0.0%	0± 0.0%
	200 mg Ni/L	0± 0.0%	0± 0.0%	0± 0.0%
nhNi@SiO_2	50 mg Ni/L	4.5± 3.8%	1.0± 0.5%	0.8± 0.1%
	100 mg Ni/L	4.8± 4.1%	0.6± 0.1%	0.6± 0.1%
	200 mg Ni/L	5.7± 6.2%	1.1± 1.0%	0.5± 0.3%
hNi@SiO_2	50 mg Ni/L	4.7± 5.1%	0.5± 0.1%	0.4± 0.1%
	100 mg Ni/L	2.5± 2.2%	0.2± 0.1%	0.2± 0.2%
	200 mg Ni/L	3.3± 1.6%	0.3± 0.2%	0.4± 0.2%
